# Sex Ratio at Birth and Mortality Rates Are Negatively Related in Humans

**DOI:** 10.1371/journal.pone.0023792

**Published:** 2011-08-24

**Authors:** Madhukar Shivajirao Dama

**Affiliations:** Rural Veterinary Dispensary, Rajola, Bidar, India; Durham University, United Kingdom

## Abstract

Evolutionary theory posits that resource availability and parental investment ability could signal offspring sex selection, in order to maximize reproductive returns. Non-human studies have provided evidence for this phenomenon, and maternal condition around the time of conception has been identified as most important factor that influence offspring sex selection. However, studies on humans have reported inconsistent results, mostly due to use of disparate measures as indicators of maternal condition. In the present study, the cross-cultural differences in human natal sex ratio were analyzed with respect to indirect measures of condition namely, life expectancy and mortality rate. Multiple regression modeling suggested that mortality rates have distinct predictive power independent of cross-cultural differences in fertility, wealth and latitude that were earlier shown to predict sex ratio at birth. These findings suggest that sex ratio variation in humans may relate to differences in parental and environmental conditions.

## Introduction

*“I formerly thought that when a tendency to produce the two sexes in equal numbers was advantageous to the species, it would follow from natural selection, but I now see that the whole problem is so intricate that it is safer to leave its solution for the future.”*

*Charles Darwin, in Descent of Man, 1874.*

Trivers and Willard predicted that, in polygynous mating systems, mothers in good condition could increase reproductive success by biasing investment in sons [1]. Superior quality sons can leave many more offspring than daughters can. Hence, where the fitness gains of offspring quality are sex specific, a female with ability to produce high-quality offspring could be expected to produce more sons and vice-versa. Empirical evidence for biased offspring sex ratios gathered from many taxa support this theory (reviewed in [2], [3]). Trivers and Willard hypothesis hold true for species that produce small litters and depends on 3 assumptions: 1) that the offspring condition at the end of parental investment is correlated with the condition of the dam; 2) that the differences in offspring condition at the end of parental investment are carried over to adulthood; and 3) that the adult will be differentially advantaged in reproductive success through slight advantages in condition.

Meta-analysis of non-human studies has suggested that sex ratio adjustments are most likely to occur around the time of conception. This adjustment was strongly correlated with maternal condition around conception, such that mothers in good condition during this period produced more sons [4]. Similar findings have been reported in humans when maternal condition was considered in relation to sex ratio adjustment [5], [6], [7], [8], [9], [10], however some studies have reported inconsistent findings [11], [12]. Further, steady decline of natal sex ratio in some countries was linked to deterioration of health due to increased exposure to environmental toxins during recent decades [13].

Average national sex ratio at birth (SRB) in humans is slightly male biased (105 males per 100 males), with remarkable deviation for some countries [14]. Systematic deviations from this ratio occurs in conditions such as economic and natural catastrophes [15], [16], war [17], [18], [19], [20], chronic stress [20], etc. Demographic factors like ethnicity [21], parental age [22], [23], mother's weight [7], birth-order [24], smoking [25], certain disease conditions [26], [27], certain professions [28], exposure to environmental toxins [29], seasonality of birth [30], etc are also linked to sex ratio adjustments. These studies have shown that higher birth-order, older parental age, low or high maternal weight, exposure to toxins and stressful events lower the chances of male births, leading to decreased SRB. While physiological basis for influence of external factors on SRB is not understood, sex hormone level alterations [31], [32], [33] and differential survival during embryogenesis [34], [35] are proposed as likely mediators.

Although, well corroborated by non-human studies, TWH still lacks support for human populations, even after identification of staggering number of factors. This is due to studies reporting conflicting results [36], [37], [38], [39], [40], [41], [42], [43], which could be attributed to the use of disparate factors like parental social status, education level, environmental calamities, resource availability, etc as measures of parental investment ability instead of lineal measures of physical condition around the time of conception [36].

Physical health and environmental conditions are directly reflected in life expectancy, the duration an individual presumes they have left to live, with those living under economic constraints, diseased state and unstable environments expecting to die sooner [44], [45], [46], [47], [48]. Further, life expectancy also accurately predict actual lifespan and mortality [49], [50]. While life expectancy is subjective response of individuals about their life to come in response to existing conditions, mortality rates are reflections of the actual conditions through which the population has lived. Hence, if an individual expects to live shorter, the reproductive investment should be biased accordingly in female offspring to enhance reproductive returns and vice-versa, in line with TWH. The possibility that the human natal sex ratio may relate to variation of life-expectancy and mortality rates has received surprisingly little attention from researchers. Indeed, only one study has investigated the relation between life-expectancy and natal sex ratio in a small sample of contemporary British women, finding that women who believed they had longer to live were more likely to have a male birth than women who thought they would live shorter [51]. In light of the above, present study was conducted to examine whether global SRB variation could be explained by cross-national differences in mortality rates.

## Methods

### Dependent variable

Counts of the sex ratio at birth for the year 2009 were taken from the Central Intelligence Agency, World Factbook [63]. Sex ratio at birth is conventionally reported as the number of males per 100 females, and this convention is employed here (mean = 105.0, median = 105.0, s.d = 2.0). Afghanistan, Bangladesh, China, India, Iran, South Korea, Pakistan, and Taiwan were excluded from the analysis owing to prevalence of son-preference and extensive practice of medical termination of female fetuses (Hesketh & Xing 2006).

### Independent variables

As a measure of mortality level for each nation, life expectancy at birth (2008, mean = 68.5, median = 71.9, s.d = 10.4) and healthy life expectancy (HALE; 2007, mean = 60.0, median = 62.0, s.d = 9.9) were used [64]. While, life expectancy at birth summarizes the mortality pattern that prevails across all age groups, healthy life expectancy (HALE) at birth adds up expectation of life for different health states and measures average number of years that a person can expect to live in “full health” by taking into account years lived in less than full health due to disease and/or injury. These two measures reflect age-standardized summary of mortality in a population, however mortality rates at different stages of life were also studied to identify if any of these have stronger correlation with SRB. Infant mortality rate (IMR, 2009, mean = 33.5, median = 19.0, s.d = 32.8), under-five mortality rate (U5MR, 2009, mean = 48.3, median = 22.0, s.d = 54.6), maternal mortality ratio (MMR, 2008, mean = 199.2, median = 65.0, s.d = 273.44) and adult mortality rate (AMR, 2008, mean = 211.2, median = 167.0, s.d = 132.3) were taken from WHO [64]. While IMR and MMR are actual number of deaths of infant (during the first year of life per 1000 live births in a given year) and mothers (per 100000 live births in a given year), U5MR and AMR are the probabilities of dying before reaching the age of five and between the age of 15 to 65 respectively. All the four variables were log transformed for normality.

### Control variables

Fertility and sex ratio at birth are negatively correlated in human populations [52]. Fertility values (2008) were taken from World Bank [65]. Total fertility rate represents the number of children that would be born to a woman if she were to live to the end of her childbearing years and bear children in accordance with current age-specific fertility rates (mean = 2.9, median = 2.4, s.d. = 1.5). Wealth and sex ratio at birth are positively correlated in humans [53]. Gross domestic product per capita based on purchasing power parity (GDP, 2009) is used as a measure of wealth. GDP data was taken from the World Factbook [63]. Values were log transformed for normality (mean = 3.7, median = 3.6, s.d. = 0.7). Latitude, which represents the angular distance of a location south or north of the equator and sex ratio at birth are positively correlated in human populations [54]. Latitude values for nations were obtained from the World Factbook [63] and numerical values were used irrespective of direction (mean = 25.9, median = 22.0, s.d. = 17.2).

### Statistical analysis

The relationship between life expectancy and sex ratio at birth was studied using regression model. Sex ratio at birth was entered as dependent variable and life expectancy was entered as independent variable. Each regression model included latitude, fertility rate and wealth as control variables. For variables showing high collinearity (square root of variance inflation factor greater than 2.0), ridge regression model was employed [55]. Ridge regression artificially reduces correlation coefficient of each pair of variables by incorporating a ridge parameter to the diagonal of a correlation matrix of highly collinear independent variables, leading to reduced error variance of estimators. Based on this principle, ridge regression overcomes the collinearity problem [56]. Continent of origin was used as a nested variable in each model to make the sex ratio data for each nation an independent datapoint and control for continental variation that may influence sex ratio trends. Eight outliers were identified (i.e., Albania, Azerbaijan, Barbados, Armenia, Georgia, Grenada, Nigeria, and United States of America) that were preventing the data from confirming to the assumptions of regression model (identified based on large standardized residual values). However, all the regression models were also constructed for a second set of data that included these outliers. Before accepting the final model, the residuals were confirmed to be homoscedastic (Breusch–Pagan test, p>0.05) and normally distributed (Ryan-Joiner's test, p>0.05). Statistical analyses were conducted using SPSS v. 16 and STATISTICA v. 10. Individual variables for all the nations are listed in table S1.

## Results

Globally, natal sex ratios were positively correlated with life expectancy and healthy life expectancy (*r* = 0.68 and 0.67, both p<0.001, table 1), demonstrating that significantly more sons are born in populations with superior life expectancy (LE: adjusted R^2^ = 0.46, β = 0.69, p<0.001, HALE: adjusted R^2^ = 0.45, β = 0.67, p<0.001). This effect was linear: addition of polynomial function of these variables i.e. LE^2^ or HALE^2^ (LE: ΔR^2^ = 0.001, p = 0.584, HALE: ΔR^2^ = 0.005, p = 0.237) to the regression model did not change the R^2^ value.

**Table 1:** Correlation among the primary variables in the study: sex ratio at birth, life expectancy at birth, healthy life expectancy, adult mortality rate, infant mortality rate, under 5 mortality rate and maternal mortality ratio.

	1	2	3	4	5	6	7	8	9	10
Sex ratio at birth	1	.679**	.669**	−.627**	−.629**	−.626**	−.639**	.494**	.516**	−.642**
Life expectancy at birth		1	.980**	−.934**	−.816**	−.898**	−.914**	.529**	.801**	−.824**
Healthy life expectancy			1	−.929**	−.823**	−.894**	−.915**	.571**	.844**	−.831**
Adult mortality rate				1	.769**	.780**	.799**	−.427**	−.720**	.690**
Maternal mortality ratio					1	.827**	.827**	−.526**	−.713**	.767**
Under 5 mortality rate						1	.988**	−.545**	−.751**	.895**
Infant mortality rate							1	−.574**	−.792**	.890**
Latitude								1	.578**	−.601**
GDP									1	−.748**
Fertility										1

**p<0.01.

Correlations are Pearson's *r*, n = 167.

**Figure 1:**
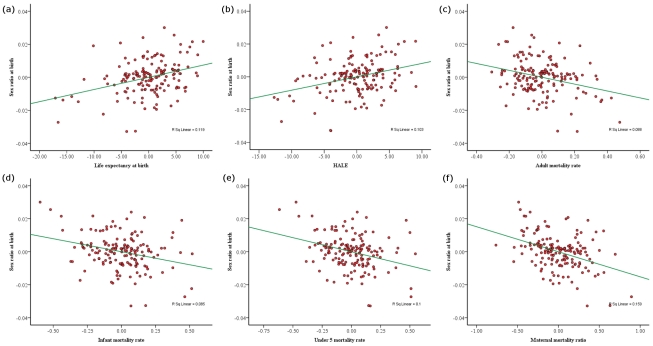
Association between (a) life expectancy at birth (b) healthy life expectancy (c) adult mortality rate (d) infant mortality rate (e) under 5 mortality rate and (f) maternal mortality ratio and sex ratio at birth, after the effects of other explanatory variables on birth sex ratio are removed (**table 2**).

In separate multiple regression models, sex ratio was predicted using different mortality rates after controlling for latitude, fertility and GDP. Life expectancy at birth and HALE were significant positive predictors and IMR, MMR, U5MR and AMR were significant negative predictors of sex ratios (table 2 and figure 1). Similar results were obtained using data consisting of statistical outliers (table S2).

**Table 2:** Multiple regression analysis predicting sex ratio at birth by life expectancy at birth (1), healthy life expectancy (2), adult mortality rate (3), infant mortality rate (4), under 5 mortality rate (5) and maternal mortality ratio (6), after controlling for fertility, wealth and latitude (n = 159).

		*β* (± s.e.)	*t*	*p*
1	Intercept		100.969	0.000
	Latitude	0.169 (0.070)	2.415	0.017
	GDP	−0.086 (0.087)	−0.980	0.329
	Fertility	−0.223 (0.093)	−2.415	0.017
	Continent	0.053 (0.067)	0.783	0.435
	Life expectancy at birth	0.402 (0.099)	4.057	0.000
R^2^ = .48, adjusted R^2^ = .46, F(5,153) = 27.86, p<.00001				
2	Intercept		106.936	0.000
	Latitude	0.155 (0.071)	2.192	0.030
	GDP	−0.101 (0.092)	−1.096	0.275
	Fertility	−0.248 (0.092)	−2.690	0.008
	Continent	0.053 (0.069)	0.775	0.440
	healthy life expectancy	0.389 (0.107)	3.621	0.000
R^2^ = .47, adjusted R^2^ = .45, F(5,153) = 26.70, p<.00001				
3	Intercept		58.277	0.000
	Latitude	0.188 (0.000)	2.472	0.015
	GDP	−0.246 (0.002)	−2.184	0.030
	Fertility	−0.387 (0.001)	−3.874	0.000
	Continent	0.073 (0.001)	1.032	0.303
	Adult mortality rate	−0.406 (0.005)	−3.839	0.000
R^2^ = .50, adjusted R^2^ = .49, F(5,153) = 31.13, p<.00001				
4	Intercept		98.130	0.000
	Latitude	0.120 (0.074)	1.617	0.108
	GDP	−0.112 (0.098)	−1.141	0.256
	Fertility	−0.280 (0.091)	−3.058	0.003
	Continent	0.105 (0.066)	1.592	0.113
	Infant mortality rate	−0.356 (0.112)	−3.171	0.002
R^2^ = .46, adjusted R^2^ = .44, F(5,153) = 25.65, p<.00001				
5	Intercept		99.330	0.000
	Latitude	0.118 (0.074)	1.599	0.112
	GDP	−0.117 (0.096)	−1.216	0.226
	Fertility	−0.251 (0.093)	−2.687	0.008
	Continent	0.096 (0.066)	1.455	0.148
	Under 5 years mortality rate	−0.393 (0.116)	−3.388	0.001
R^2^ = .46, adjusted R^2^ = .44, F(5,153) = 26.14, p<.00001				
6	Intercept		103.360	0.000
	Latitude	0.061 (0.075)	0.807	0.421
	GDP	−0.142 (0.092)	−1.538	0.126
	Fertility	−0.244 (0.089)	−2.733	0.007
	Continent	0.106 (0.064)	1.650	0.101
	Maternal mortality ratio	−0.473 (0.110)	−4.305	0.000
R^2^ = .48, adjusted R^2^ = .47, F(5,153) = 28.58, p<.00001				

Except (3), all are multiple ridge regression models at λ = 0.1, see methods for details.

## Discussion

The correlation between mortality indices and SRB was statistically significant at the national level worldwide. All the analysis showed that mortality rates were a significant predictors of sex ratios, whether using either individual mortality rates (IMR, MMR, U5MR and AMR) or averaged mortality indices (life expectancy and HALE). The zero-order correlation between life expectancy and sex ratio was higher than that of any other variable for which there is a previously proposed correlation (Table 1).

Multiple regression shows that, life expectancy and mortality rates have distinct predictive power beyond fertility, wealth and latitude. The correlation among SRB, fertility, GDP and life expectancy suggests that as human populations become more wealthy, the life expectancy increases (*r* = 0.80, p<0.001), total fertility is reduced (*r* = −0.75, p<0.001) and more sons are produced (*r* = 0.52, p<0.001) (table 1). Although the effect of GDP is not statistically significant when other factors are present, it cannot be said that GDP is not involved. A nation with higher average GDP will also be able to afford better education and medical services, leading to reduced mortality and extended life expectancy, which may indirectly lead to increased son births, by enhancing the parental investment ability. These sources of endogeneity must be considered when interpreting present results. It should also be noted that, global variation in sex ratios is not solely related to life expectancy and mortality rate, rather, it is probably influenced by a variety of factors, including those mentioned here as well as factors that are yet unknown.

Life expectancy and mortality rates impose substantial influence on reproductive [57] and parental investment strategies in humans [58]. Unfortunately, demographic data do not allow us to distinguish sex-specific embryonic mortality, parental hormonal alterations around conception or other proximate mechanisms that may underlie the relationship between mortality rates and sex ratios. However, in the absence of known physiological mechanism, increase in testosterone level that occurs under favorable environmental conditions [59], [60], which is linked with more male births [61], might be a probable basis. Alternatively, the relation may be completely non-adaptive and outcome of various reproductive constraints and differential embryonic deaths [62], that occur in unfavorable environments. Adaptive or otherwise, the human natal sex ratio differences across the world may be influenced by existing environmental conditions and perceived future survival, as shown by the above results.

## Supporting Information

Table S1 **Descriptive statistics for countries included in this study.**
(DOCX)

Table S2 **Multiple regression analysis predicting sex ratio at birth by life expectancy at birth (1), healthy life expectancy (2), adult mortality rate (3), infant mortality rate (4), under 5 mortality rate (5) and maternal mortality ratio (6), after controlling for fertility, wealth and latitude (n = 167).** Except (3), all are ridge regression models at λ = 0.1, see methods for details. This analysis includes statistical outliers that were excluded from earlier regression models (Table 2).
(DOCX)
