# Supplementary material for: Sex Ratio at Birth and Mortality Rates Are Negatively Related in Humans
Source: PLoS One. 2011 Aug 24;6(8):e23792. doi: 10.1371/journal.pone.0023792 (PMC3161077; doi:10.1371/journal.pone.0023792)
Supplement: Table S1 — Descriptive statistics for countries included in this study. (DOCX) [file pone.0023792.s001.docx]

Table S1. Descriptive statistics for countries included in this study.

| Country | Adult mortality rate | Life expectancy at birth | Maternal mortality ratio | Under 5 mortality rate | Infant mortality rate | Healthy life expectancy | Sex ratio at birth | Latitude | logGDP | fertility |
| --- | --- | --- | --- | --- | --- | --- | --- | --- | --- | --- |
| Albania | 116 | 76.63371 | 31 | 14 | 13 | 64 | 110 | 41 | 3.57 | 1.86 |
| Algeria | 132 | 72.38905 | 120 | 41 | 36 | 62 | 105 | 28 | 3.61 | 2.36 |
| Angola | 421 | 47.03773 | 610 | 220 | 130 | 45 | 105 | 12.5 | 3.57 | 5.76 |
| AntiguaandBarbuda | 176 | 75.48 | 150 | 12 | 11 | 66 | 105 | 17.03 | 4.22 | 2.05 |
| Argentina | 123 | 75.33398 | 70 | 15 | 13 | 67 | 105 | 34 | 3.88 | 2.24 |
| Armenia | 165 | 73.53739 | 29 | 23 | 21 | 61 | 114 | 40 | 3.45 | 1.74 |
| Australia | 63 | 81.39512 | 8 | 5 | 4 | 74 | 106 | 27 | 4.63 | 1.97 |
| Austria | 75 | 80.44756 | 5 | 4 | 4 | 72 | 105 | 47.3 | 4.66 | 1.41 |
| Azerbaijan | 182 | 70.17768 | 38 | 36 | 32 | 59 | 113 | 40.5 | 3.69 | 2.3 |
| Bahamas | 166 | 73.48546 | 49 | 13 | 9 | 65 | 102 | 24.3 | 4.34 | 2.01 |
| Bahrain | 103 | 75.91149 | 19 | 12 | 10 | 66 | 103 | 26 | 4.45 | 2.27 |
| Barbados | 138 | 77.0089 | 64 | 11 | 10 | 67 | 101 | 13.2 | 4.16 | 1.53 |
| Belarus | 221 | 70.63288 | 15 | 13 | 11 | 62 | 106 | 53 | 3.7 | 1.42 |
| Belgium | 86 | 80.10956 | 5 | 5 | 4 | 72 | 104 | 50.8 | 4.64 | 1.82 |
| Belize | 178 | 76.32329 | 94 | 19 | 17 | 60 | 105 | 17.3 | 3.63 | 2.9 |
| Benin | 301 | 61.37798 | 410 | 121 | 76 | 50 | 105 | 9.5 | 2.87 | 5.45 |
| Bhutan | 229 | 66.13356 | 200 | 81 | 54 | 55 | 105 | 27.5 | 3.26 | 2.64 |
| Bolivia | 196 | 65.68378 | 180 | 54 | 46 | 58 | 105 | 17 | 3.25 | 3.46 |
| BosniaandHerzegovina | 107 | 75.10632 | 9 | 15 | 13 | 67 | 107 | 44 | 3.66 | 1.21 |
| Botswana | 404 | 54.24102 | 190 | 31 | 26 | 49 | 103 | 22 | 3.78 | 2.87 |
| Brazil | 158 | 72.40212 | 58 | 22 | 18 | 64 | 105 | 10 | 3.91 | 1.88 |
| BruneiDarussalam | 94 | 77.3641 | 21 | 7 | 5 | 66 | 105 | 4.5 | 4.48 | 2.08 |
| Bulgaria | 153 | 73.31659 | 13 | 11 | 9 | 66 | 106 | 43 | 3.79 | 1.48 |
| BurkinaFaso | 372 | 52.98768 | 560 | 169 | 92 | 43 | 103 | 13 | 2.71 | 5.91 |
| Burundi | 411 | 50.43366 | 970 | 168 | 102 | 43 | 103 | 3.5 | 2.2 | 4.59 |
| Cambodia | 253 | 60.96851 | 290 | 89 | 69 | 53 | 104 | 13 | 2.83 | 2.91 |
| Cameroon | 403 | 51.06437 | 600 | 131 | 82 | 45 | 103 | 6 | 3.05 | 4.62 |
| Canada | 70 | 80.96488 | 12 | 6 | 5 | 73 | 106 | 60 | 4.6 | 1.6 |
| CapeVerde | 185 | 71.04037 | 94 | 29 | 24 | 61 | 103 | 16 | 3.49 | 2.73 |
| CentralAfricanRepublic | 457 | 46.95861 | 850 | 173 | 115 | 42 | 103 | 7 | 2.66 | 4.8 |
| Chad | 446 | 48.73061 | 1200 | 209 | 124 | 40 | 104 | 15 | 2.78 | 6.16 |
| Chile | 88 | 78.61395 | 26 | 9 | 7 | 70 | 105 | 30 | 3.98 | 1.93 |
| Colombia | 118 | 72.98049 | 85 | 20 | 16 | 66 | 103 | 4 | 3.7 | 2.43 |
| Comoros | 258 | 65.34573 | 340 | 105 | 75 | 56 | 103 | 12.2 | 2.92 | 3.95 |
| Congo | 381 | 53.55188 | 580 | 127 | 80 | 48 | 103 | 1 | 3.62 | 5.68 |
| CostaRica | 97 | 78.91802 | 44 | 11 | 10 | 69 | 105 | 10 | 3.8 | 1.96 |
| Côted'Ivoire | 361 | 57.44244 | 470 | 114 | 81 | 47 | 103 | 8 | 3.04 | 4.6 |
| Croatia | 115 | 75.9122 | 14 | 5 | 4 | 68 | 106 | 45.2 | 4.15 | 1.47 |
| Cuba | 102 | 78.72134 | 53 | 6 | 5 | 69 | 106 | 21.5 | 3.99 | 1.51 |
| Cyprus | 61 | 79.66149 | 10 | 4 | 4 | 70 | 105 | 35 | 4.5 | 1.52 |
| CzechRepublic | 105 | 77.21122 | 8 | 4 | 3 | 70 | 106 | 49.8 | 4.26 | 1.5 |
| Denmark | 90 | 78.70049 | 5 | 4 | 4 | 72 | 106 | 56 | 4.75 | 1.89 |
| Djibouti | 309 | 55.38924 | 300 | 95 | 76 | 48 | 103 | 11.5 | 3.08 | 3.9 |
| Dominica | 164 | 75.98 | 65 | 10 | 9 | 66 | 105 | 15.25 | 4.02 | 2.07 |
| DominicanRepublic | 158 | 72.57076 | 100 | 33 | 27 | 63 | 104 | 19 | 3.66 | 2.65 |
| Ecuador | 165 | 75.13463 | 140 | 25 | 21 | 64 | 105 | 2 | 3.62 | 2.56 |
| Egypt | 187 | 70.13824 | 82 | 23 | 20 | 60 | 105 | 27 | 3.36 | 2.86 |
| ElSalvador | 214 | 71.26051 | 110 | 18 | 16 | 61 | 105 | 13.8 | 3.56 | 2.32 |
| EquatorialGuinea | 361 | 50.22749 | 280 | 147 | 90 | 46 | 103 | 2 | 4.19 | 5.34 |
| Eritrea | 228 | 59.45222 | 280 | 58 | 41 | 55 | 103 | 15 | 2.53 | 4.63 |
| Estonia | 165 | 73.97317 | 12 | 6 | 5 | 66 | 106 | 59 | 4.15 | 1.66 |
| Ethiopia | 307 | 55.195 | 470 | 109 | 69 | 50 | 103 | 8 | 2.54 | 5.32 |
| Fiji | 204 | 68.86573 | 26 | 18 | 16 | 62 | 105 | 18 | 3.55 | 2.73 |
| Finland | 94 | 79.79195 | 8 | 3 | 3 | 72 | 104 | 64 | 4.65 | 1.85 |
| France | 87 | 81.52049 | 8 | 4 | 3 | 73 | 105 | 46 | 4.61 | 2 |
| Gabon | 327 | 60.44393 | 260 | 77 | 57 | 52 | 103 | 1 | 3.88 | 3.31 |
| Gambia | 276 | 55.93239 | 400 | 106 | 80 | 51 | 103 | 13.5 | 2.63 | 5.05 |
| Georgia | 157 | 71.54812 | 48 | 30 | 26 | 64 | 113 | 42 | 3.39 | 1.58 |
| Germany | 78 | 80.08854 | 7 | 4 | 4 | 73 | 106 | 51 | 4.61 | 1.38 |
| Ghana | 273 | 56.61695 | 350 | 76 | 51 | 50 | 103 | 8 | 2.82 | 4 |
| Greece | 75 | 79.96317 | 2 | 3 | 3 | 72 | 106 | 39 | 4.47 | 1.51 |
| Grenada | 228 | 75.32178 | 1 | 15 | 13 | 61 | 110 | 12.07 | 4.02 | 2.18 |
| Guatemala | 228 | 70.33502 | 110 | 34 | 29 | 60 | 105 | 15.5 | 3.42 | 4.11 |
| Guinea | 336 | 57.82124 | 680 | 146 | 90 | 47 | 103 | 11 | 2.59 | 5.41 |
| Guinea-Bissau | 403 | 47.82344 | 1000 | 195 | 117 | 42 | 103 | 12 | 2.46 | 5.71 |
| Guyana | 261 | 67.10507 | 270 | 61 | 46 | 53 | 105 | 5 | 3.18 | 2.32 |
| Haiti | 267 | 61.21466 | 300 | 72 | 54 | 54 | 103 | 19 | 2.82 | 3.5 |
| Honduras | 179 | 72.19605 | 110 | 31 | 26 | 62 | 105 | 15 | 3.29 | 3.26 |
| Hungary | 167 | 74.00902 | 13 | 7 | 6 | 66 | 106 | 47 | 4.11 | 1.35 |
| Iceland | 56 | 81.57512 | 5 | 3 | 2 | 74 | 104 | 65 | 4.58 | 2.14 |
| Indonesia | 206 | 70.79329 | 240 | 41 | 31 | 60 | 105 | 5 | 3.37 | 2.17 |
| Iraq | 285 | 67.92966 | 75 | 45 | 36 | 54 | 105 | 33 | 3.32 | 4.05 |
| Ireland | 73 | 79.85683 | 3 | 5 | 4 | 73 | 107 | 53 | 4.71 | 2.1 |
| Israel | 66 | 81.00244 | 7 | 5 | 4 | 73 | 105 | 31.5 | 4.42 | 2.96 |
| Italy | 61 | 81.94521 | 5 | 4 | 3 | 74 | 107 | 42.8 | 4.55 | 1.41 |
| Jamaica | 175 | 71.84315 | 89 | 31 | 26 | 64 | 105 | 18.3 | 3.74 | 2.39 |
| Japan | 65 | 82.58756 | 6 | 3 | 3 | 76 | 106 | 36 | 4.6 | 1.34 |
| Jordan | 149 | 72.71356 | 59 | 20 | 17 | 63 | 106 | 31 | 3.58 | 3.49 |
| Kazakhstan | 310 | 66.44215 | 45 | 30 | 27 | 56 | 106 | 48 | 3.84 | 2.56 |
| Kenya | 371 | 54.23744 | 530 | 128 | 81 | 48 | 102 | 1 | 2.88 | 4.92 |
| Kuwait | 61 | 77.96963 | 9 | 11 | 9 | 69 | 104 | 29.3 | 4.73 | 2.17 |
| Kyrgyzstan | 264 | 67.36976 | 81 | 38 | 33 | 57 | 105 | 41 | 2.93 | 2.7 |
| LaoS | 302 | 64.97295 | 580 | 61 | 48 | 54 | 105 | 18 | 2.97 | 3.47 |
| Latvia | 213 | 72.23829 | 20 | 9 | 8 | 64 | 105 | 57 | 4.07 | 1.45 |
| Lebanon | 160 | 72.04732 | 26 | 13 | 12 | 62 | 105 | 33.8 | 3.91 | 1.85 |
| Lesotho | 685 | 44.99493 | 530 | 79 | 63 | 40 | 103 | 29.5 | 2.93 | 3.33 |
| Liberia | 340 | 58.26312 | 990 | 144 | 100 | 48 | 103 | 6.5 | 2.35 | 5.9 |
| Libya | 138 | 74.329 | 64 | 17 | 15 | 64 | 105 | 25 | 3.99 | 2.7 |
| Lithuania | 215 | 71.82171 | 13 | 7 | 5 | 63 | 106 | 56 | 4.05 | 1.47 |
| Luxembourg | 79 | 80.52463 | 17 | 3 | 2 | 73 | 107 | 49.8 | 5.02 | 1.61 |
| Macedonia | 116 | 74.21132 | 9 | 11 | 10 | 66 | 108 | 41.5 | 3.97 | 1.58 |
| Madagascar | 263 | 60.34149 | 440 | 106 | 68 | 52 | 103 | 20 | 2.66 | 4.72 |
| Malawi | 481 | 53.0591 | 510 | 100 | 65 | 44 | 102 | 13.5 | 2.51 | 5.55 |
| Malaysia | 139 | 74.37971 | 31 | 6 | 6 | 64 | 107 | 2.5 | 3.84 | 2.56 |
| Maldives | 86 | 71.58212 | 37 | 28 | 24 | 64 | 105 | 3.3 | 3.64 | 2.02 |
| Mali | 386 | 48.42902 | 830 | 194 | 102 | 42 | 103 | 17 | 2.84 | 6.54 |
| Malta | 61 | 79.64098 | 8 | 7 | 7 | 72 | 106 | 35.8 | 4.26 | 1.43 |
| Mauritania | 290 | 56.72883 | 550 | 118 | 75 | 51 | 103 | 20 | 2.96 | 4.47 |
| Mauritius | 160 | 72.57073 | 36 | 16 | 14 | 63 | 105 | 20.3 | 3.83 | 1.58 |
| Mexico | 121 | 75.06545 | 85 | 17 | 15 | 67 | 105 | 23 | 3.91 | 2.1 |
| Moldova | 227 | 68.43715 | 32 | 17 | 15 | 61 | 106 | 47 | 3.4 | 1.29 |
| Mongolia | 219 | 66.56941 | 65 | 41 | 33 | 58 | 105 | 46 | 3.2 | 2 |
| Montenegro | 131 | 74.09754 | 15 | 9 | 8 | 65 | 107 | 42.3 | 4 | 1.96 |
| Morocco | 118 | 71.29178 | 110 | 36 | 32 | 62 | 105 | 32 | 3.45 | 2.35 |
| Mozambique | 470 | 47.89446 | 550 | 130 | 90 | 42 | 102 | 18.3 | 2.63 | 5.06 |
| Myanmar | 336 | 61.55485 | 240 | 122 | 76 | 50 | 106 | 22 | 3.04 | 2.3 |
| Namibia | 320 | 61.01359 | 180 | 42 | 31 | 52 | 103 | 22 | 3.64 | 3.36 |
| Nepal | 277 | 66.69007 | 380 | 51 | 41 | 55 | 104 | 28 | 2.63 | 2.9 |
| Netherlands | 68 | 80.40073 | 9 | 5 | 4 | 73 | 105 | 52.5 | 4.68 | 1.78 |
| NewZealand | 72 | 80.15122 | 14 | 6 | 5 | 73 | 105 | 41 | 4.46 | 2.2 |
| Nicaragua | 165 | 73.142 | 100 | 27 | 23 | 64 | 105 | 13 | 3.04 | 2.72 |
| Niger | 359 | 51.40146 | 820 | 167 | 79 | 44 | 103 | 16 | 2.55 | 7.12 |
| Nigeria | 411 | 47.90859 | 840 | 186 | 96 | 42 | 106 | 10 | 3.04 | 5.7 |
| Norway | 67 | 80.74146 | 7 | 3 | 3 | 73 | 105 | 62 | 4.9 | 1.96 |
| Oman | 129 | 75.91322 | 20 | 12 | 10 | 65 | 105 | 21 | 4.34 | 3.05 |
| Panama | 112 | 75.66076 | 71 | 23 | 19 | 67 | 104 | 9 | 3.85 | 2.55 |
| PapuaNewGuinea | 264 | 61.10102 | 250 | 69 | 53 | 56 | 105 | 6 | 3.07 | 4.07 |
| Paraguay | 139 | 71.88478 | 95 | 28 | 24 | 64 | 105 | 23 | 3.37 | 3.05 |
| Peru | 106 | 73.2619 | 98 | 24 | 22 | 67 | 105 | 10 | 3.64 | 2.57 |
| Philippines | 174 | 71.83446 | 94 | 32 | 26 | 62 | 105 | 13 | 3.24 | 3.08 |
| Poland | 142 | 75.53317 | 6 | 7 | 6 | 67 | 106 | 52 | 4.05 | 1.39 |
| Portugal | 90 | 79.24976 | 7 | 4 | 3 | 71 | 107 | 39.5 | 4.33 | 1.37 |
| Qatar | 72 | 75.9421 | 8 | 8 | 7 | 67 | 106 | 25.5 | 4.8 | 2.41 |
| Romania | 156 | 73.37341 | 27 | 13 | 11 | 65 | 106 | 46 | 3.88 | 1.35 |
| RussianFederation | 273 | 67.84463 | 39 | 11 | 9 | 60 | 106 | 60 | 3.94 | 1.49 |
| Rwanda | 303 | 50.13093 | 540 | 112 | 72 | 43 | 103 | 2 | 2.7 | 5.41 |
| SaintKittsandNevis | 138 | 74.6 | 130 | 15 | 14 | 64 | 102 | 17.2 | 4.16 | 1.79 |
| SaintLucia | 144 | 76.84 | 30 | 15 | 13 | 66 | 106 | 13.53 | 4.05 | 1.81 |
| SaudiArabia | 154 | 73.11905 | 24 | 21 | 18 | 62 | 105 | 25 | 4.16 | 3.12 |
| Senegal | 269 | 55.59529 | 410 | 108 | 57 | 51 | 103 | 14 | 3.02 | 4.82 |
| Serbia | 138 | 73.63659 | 8 | 8 | 7 | 65 | 107 | 44 | 3.76 | 1.4 |
| SierraLeone | 393 | 47.59998 | 970 | 194 | 123 | 35 | 103 | 8.5 | 2.53 | 5.2 |
| Singapore | 64 | 80.74146 | 9 | 3 | 2 | 73 | 108 | 1.4 | 4.56 | 1.28 |
| Slovakia | 135 | 74.81073 | 6 | 7 | 6 | 67 | 105 | 48.7 | 4.21 | 1.32 |
| Slovenia | 95 | 78.9739 | 18 | 3 | 2 | 71 | 107 | 46 | 4.38 | 1.53 |
| SolomonIslands | 160 | 66.25502 | 100 | 36 | 30 | 59 | 105 | 8 | 3.1 | 3.87 |
| Somalia | 416 | 49.83544 | 1200 | 200 | 119 | 45 | 103 | 10 | 2.78 | 6.39 |
| SouthAfrica | 520 | 51.47724 | 410 | 67 | 48 | 48 | 102 | 29 | 3.76 | 2.54 |
| Spain | 72 | 81.08805 | 6 | 4 | 4 | 74 | 107 | 40 | 4.5 | 1.46 |
| SriLanka | 209 | 74.12712 | 39 | 17 | 13 | 63 | 104 | 7 | 3.32 | 2.33 |
| Sudan | 319 | 58.14785 | 750 | 109 | 70 | 50 | 105 | 15 | 3.11 | 4.17 |
| Suriname | 174 | 69.02151 | 100 | 27 | 25 | 61 | 107 | 4 | 3.77 | 2.4 |
| Swaziland | 620 | 45.7652 | 420 | 83 | 59 | 42 | 103 | 26.5 | 3.39 | 3.53 |
| Sweden | 62 | 81.23717 | 5 | 3 | 2 | 74 | 106 | 62 | 4.64 | 1.91 |
| Switzerland | 60 | 82.16171 | 10 | 5 | 4 | 75 | 105 | 47 | 4.82 | 1.48 |
| SyrianArabRepublic | 150 | 74.22532 | 46 | 16 | 14 | 63 | 106 | 35 | 3.39 | 3.25 |
| Tajikistan | 173 | 66.74702 | 64 | 64 | 54 | 57 | 105 | 39 | 2.85 | 3.41 |
| Tanzania | 458 | 55.6468 | 790 | 103 | 67 | 45 | 103 | 6 | 3.18 | 4.16 |
| Thailand | 209 | 68.86012 | 48 | 14 | 13 | 62 | 105 | 15 | 3.59 | 1.82 |
| Timor-Leste | 240 | 61.12141 | 370 | 93 | 75 | 53 | 105 | 8.5 | 2.69 | 6.48 |
| Togo | 323 | 62.50746 | 350 | 98 | 64 | 51 | 103 | 8 | 2.63 | 4.26 |
| TrinidadandTobago | 163 | 69.33927 | 55 | 35 | 31 | 62 | 103 | 11 | 4.2 | 1.64 |
| Tunisia | 103 | 74.30244 | 60 | 21 | 18 | 66 | 107 | 34 | 3.58 | 2.06 |
| Turkey | 106 | 71.89154 | 23 | 22 | 20 | 66 | 105 | 39 | 3.92 | 2.11 |
| Turkmenistan | 296 | 64.82122 | 77 | 48 | 43 | 55 | 105 | 40 | 3.59 | 2.48 |
| Uganda | 436 | 52.66678 | 430 | 135 | 84 | 42 | 103 | 1 | 2.68 | 6.34 |
| Ukraine | 277 | 68.25146 | 26 | 15 | 14 | 60 | 106 | 49 | 3.39 | 1.39 |
| UnitedArabEmirates | 73 | 77.74807 | 10 | 8 | 7 | 68 | 105 | 24 | 4.77 | 1.94 |
| UnitedKingdom | 78 | 79.90337 | 12 | 6 | 5 | 72 | 105 | 54 | 4.55 | 1.94 |
| UnitedStates | 107 | 78.43902 | 24 | 8 | 7 | 70 | 113 | 38 | 4.67 | 2.1 |
| Uruguay | 121 | 75.98073 | 27 | 16 | 14 | 67 | 104 | 33 | 4.03 | 2.01 |
| Uzbekistan | 181 | 67.75612 | 30 | 38 | 34 | 59 | 106 | 41 | 3.07 | 2.56 |
| Venezuela | 146 | 73.54907 | 68 | 18 | 16 | 66 | 105 | 8 | 4.06 | 2.54 |
| Vietnam | 150 | 74.37146 | 56 | 14 | 12 | 64 | 107 | 16 | 3.02 | 2.06 |
| Yemen | 217 | 62.91644 | 210 | 69 | 53 | 54 | 105 | 15 | 3.05 | 5.22 |
| Zambia | 515 | 45.39576 | 470 | 148 | 92 | 40 | 103 | 15 | 2.99 | 5.83 |
| Zimbabwe | 772 | 44.21441 | 790 | 96 | 62 | 39 | 103 | 19 | 2.6 | 3.43 |
