# Supplementary material for: Sex Ratio at Birth and Mortality Rates Are Negatively Related in Humans
Source: PLoS One. 2011 Aug 24;6(8):e23792. doi: 10.1371/journal.pone.0023792 (PMC3161077; doi:10.1371/journal.pone.0023792)
Supplement: Table S2 — Multiple regression analysis predicting sex ratio at birth by life expectancy at birth (1), healthy life expectancy (2), adult mortality rate (3), infant mortality rate (4), under 5 mortality rate (5) and maternal mortality ratio (6), after controlling for fertility, wealth and latitude (n = 167). Except (3), all are ridge regression models at λ = 0.1, see methods for details. This analysis includes statistical outliers that were excluded from earlier regression models (Table 2). (DOCX) [file pone.0023792.s002.docx]

Table S2. Multiple regression analysis predicting sex ratio at birth by life expectancy at birth (1), healthy life expectancy (2), adult mortality rate (3), infant mortality rate (4), under 5 mortality rate (5) and maternal mortality ratio (6), after controlling for fertility, wealth and latitude (n = 167). Except (3), all are ridge regression models at λ = 0.1, see methods for details .

|  |  | *β* (± s.e.) | *t* | *p* |
| --- | --- | --- | --- | --- |
| 1 | Intercept |  | 61.870 | 0.000 |
|  | Latitude | 0.191 (± 0.079) | 2.422 | 0.017 |
|  | GDP | -0.127 (± 0.098) | -1.300 | 0.196 |
|  | Fertility | -0.199 (± 0.105) | -1.888 | 0.061 |
|  | Continent | 0.010 (± 0.077) | 0.132 | 0.895 |
|  | Life expectancy at birth | 0.305 (± 0.113) | 2.698 | 0.008 |
| R² = .29, adjusted R² = .27, F(5,161) = 13.22, p<.00001 | | | | |
| 2 | Intercept |  | 66.066 | 0.000 |
|  | Latitude | 0.188 (± 0.080) | 2.333 | 0.021 |
|  | GDP | -0.110 (± 0.103) | -1.063 | 0.290 |
|  | Fertility | -0.245 (± 0.105) | -2.329 | 0.021 |
|  | Continent | 0.026 (± 0.079) | 0.333 | 0.740 |
|  | healthy life expectancy | 0.221 (± 0.122) | 1.809 | 0.072 |
| R² = .27, adjusted R² = .25, F(5,161) = 12.14, p<.00001 | | | | |
| 3 | Intercept |  | 35.805 | 0.000 |
|  | Latitude | 0.213 (± 0.000) | 2.443 | 0.016 |
|  | GDP | -0.228 (± 0.004) | -1.815 | 0.071 |
|  | Fertility | -0.347 (± 0.002) | -3.040 | 0.003 |
|  | Continent | 0.033 (± 0.001) | 0.411 | 0.682 |
|  | Adult mortality rate | -0.247 (± 0.009) | -2.051 | 0.042 |
| R² = .30, adjusted R² = .27, F(5,161) = 13.48, p<.00001 | | | | |
| 4 | Intercept |  | 59.412 | 0.000 |
|  | Latitude | -0.064 (± 0.111) | -0.576 | 0.565 |
|  | GDP | -0.294 (± 0.103) | -2.855 | 0.005 |
|  | Fertility | 0.064 (± 0.076) | 0.846 | 0.399 |
|  | Continent | -0.091 (± 0.127) | -0.714 | 0.476 |
|  | Infant mortality rate | 0.191 (± 0.084) | 2.279 | 0.024 |
| R² = .26, adjusted R² = .24, F(5,161) = 11.40, p<.00001 | | | | |
| 5 | Intercept |  | 60.210 | 0.000 |
|  | Latitude | -0.079 (± 0.109) | -0.725 | 0.470 |
|  | GDP | -0.277 (± 0.106) | -2.617 | 0.010 |
|  | Fertility | 0.059 (± 0.076) | 0.772 | 0.441 |
|  | Continent | -0.131 (± 0.132) | -0.995 | 0.321 |
|  | Under 5 years mortality rate | 0.185 (± 0.084) | 2.215 | 0.028 |
| R² = .26, adjusted R² = .24, F(5,161) = 11.52, p<.00001 | | | | |
| 6 | Intercept |  | 66.573 | 0.000 |
|  | Latitude | -0.184 (± 0.102) | -1.808 | 0.072 |
|  | GDP | -0.208 (± 0.100) | -2.066 | 0.040 |
|  | Fertility | 0.046 (± 0.073) | 0.629 | 0.530 |
|  | Continent | -0.389 (± 0.119) | -3.274 | 0.001 |
|  | Maternal mortality ratio | 0.110 (± 0.083) | 1.320 | 0.189 |
| R² = .31, adjusted R² = .28, F(5,161) = 14.15, p<.00001 | | | | |
